# Revealing Landscape of Competing Endogenous RNA Networks in Sepsis-Induced Cardiovascular Diseases

**DOI:** 10.31083/j.rcm2407214

**Published:** 2023-07-24

**Authors:** Wei Xiong, Shiyan Feng, Yanhua Zhao, Xinquan Liu, Jian Gong

**Affiliations:** ^1^Laboratory of Clinical Research, Ziyang People’s Hospital, Ziyang Hospital of Sichuan Provincial People’s Hospital, 641300 Ziyang, Sichuan, China; ^2^Department of Anesthesiology, West China Hospital, Sichuan University, 610041 Chengdu, Sichuan, China; ^3^Emergency Medical Center, Sichuan Provincial People’s Hospital, University of Electronic Science and Technology, 610072 Chengdu, Sichuan, China; ^4^Department of Emergency Critical Care, Ziyang People’s Hospital, Ziyang Hospital of Sichuan Provincial People’s Hospital, 641300 Ziyang, Sichuan, China

**Keywords:** cardiovascular, sepsis, competing endogenous RNA, long noncoding RNA, circular RNA, microRNA

## Abstract

Cardiovascular dysfunction induced by sepsis is one of the most common phenotypes of cardiovascular diseases (CVDs), which is closely related to the high mortality of sepsis and is an urgent health problem to be solved worldwide. Unfortunately, the exact pathogenesis and pathophysiology of sepsis-induced cardiovascular dysfunction are not clear. As a research hotspot in recent years, competing endogenous RNA (ceRNA) networks are involved in the modulation of the pathophysiological progression of many diseases, including sepsis-related CVDs. Both long noncoding RNAs (lncRNAs) and circular RNAs (circRNAs) can specifically bind to microRNAs (miRNAs) as ceRNAs to target messenger RNAs (mRNAs), forming a ceRNA network composed of lncRNA/circRNA-miRNA-mRNA. This review demonstrates the potential regulatory mechanism of the ceRNA networks in sepsis-induced cardiovascular toxicity, hoping to provide novel therapeutic strategies and monitoring targets for sepsis-related CVDs.

## 1. Introduction 

**Fig. 1.**
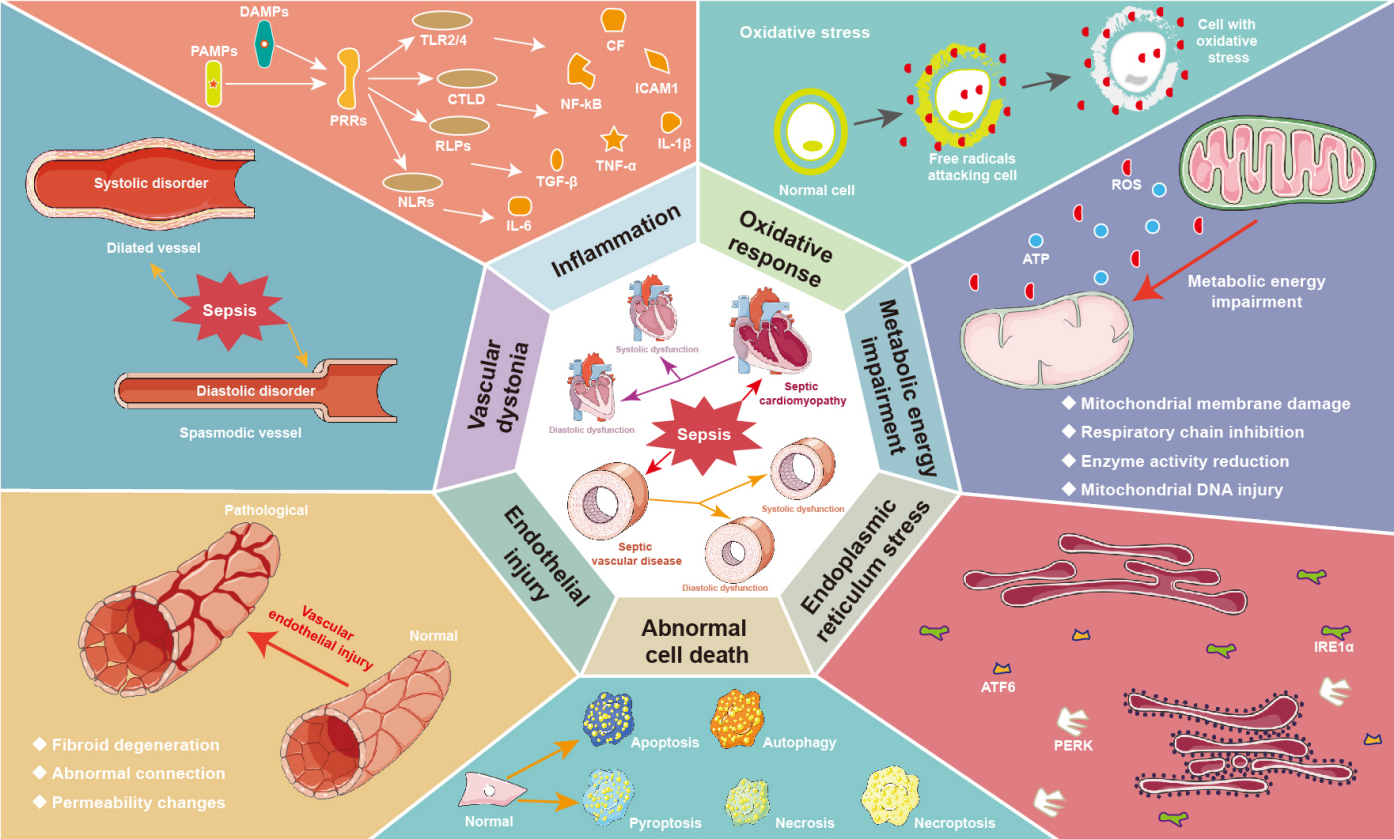
**Pathophysiological mechanism of septic cardiovascular diseases**. Septic toxicity often causes systolic and diastolic dysfunction of the heart and vessels. The main mechanisms of septic cardiomyopathy include excessive inflammation, oxidative response, metabolic energy impairment, endoplasmic reticulum stress, and abnormal cell death. In addition, sepsis induced vascular dysfunction is associated with glycocalyx damage, endothelial injury, and vascular dystonia, leading to vascular paralysis and microcirculation disturbances. ATF6, activating transcription factor 6; ATP, adenosine 5’-triphosphate; CF, cell factor; CTLD, C-type lectin-like domain; DAMPs, danger-associated molecular patterns; ICAM1, intercellular cell adhesion molecule-1; IL, interleukin; IRE1α, inositol-requiring kinase 1α; NF-κB, nuclear factor-κB; NLRs, NOD-like receptors; PAMPs, pathogen-associated molecular patterns; PERK, protein kinase R-like endoplasmic reticulum kinase; PRRs, pattern recognition receptors; ROS, reactive oxygen species; TGF-β, transforming growth factor-β; TLR, toll-like receptor; TNF-α, tumor necrosis factor-α.

Sepsis is a syndrome of th systemic inflammatory response caused by infection and, ultimately, multiorgan dysfunction [1], which endangers millions of patients worldwide each year and has high mortality rates ranging from one-in-six to one-in-three [2]. The cardiovascular system has been considered as the most frequently affected organ system during sepsis and plays a crucial role in the pathophysiology of septic organ dysfunction. Cardiac depression caused by sepsis is a common phenotype in septic cardiomyopathy and suggests a poor clinical prognosis. Septic cardiomyopathy is characterized by reversible systolic and diastolic dysfunction of the heart throughout the cardiac cycle under septic conditions, which involves complex responses to pathogens, excessive inflammation, oxidative response, metabolic energy impairment, endoplasmic reticulum (ER) stress, myocardial apoptosis and structural changes [3], as shown in Fig. 1. In addition, vascular dysfunction has been recognized as the other common phenotype in sepsis, and is associated with glycocalyx damage, endothelial injury, and vascular dystonia, leading to vascular paralysis, microcirculation disturbances, and septic shock [4]. Septic shock is usually characterized by fluid resuscitation-refractory hypotension and hyperlactatemia. With the development of training, monitoring and treatment in intensive care units, the hospital mortality of septic shock has dropped from 80% to 30%, but this condition is still life-threatening [5].

Sepsis-induced cardiovascular diseases (SCVDs, a classic form of CVD) have been confirmed to be the major reason for the increased mortality in patients with sepsis and septic shock, which are considered serious healthcare problems [6]. Despite significant advances in anti-infection and organ supportive therapy, sepsis-associated death remains high. Even survivors who suffer from severe sepsis are often left with long-term sequelae and higher recurrence rates, resulting in a huge social, economic and public health burden [7]. A great number of studies on septic cardiovascular dysfunction have been carried out in recent decades, but the exact pathophysiology and pathogenesis are still unclear. Sepsis lacks ideal biomarkers and has no specific treatment beyond infection control and symptomatic support [8]. Early diagnosis and bundled treatment of sepsis within the first few hours can improve long-term outcomes in SCVDs [9]. Therefore, biomarkers for early detection and precision therapy are urgently needed to improve the survival rate and living quality of patients with SCVDs. Elucidating the molecular mechanism of cardiovascular dysfunction induced by sepsis can provide novel monitoring and therapeutic targets for SCVDs.

**Fig. 2.**
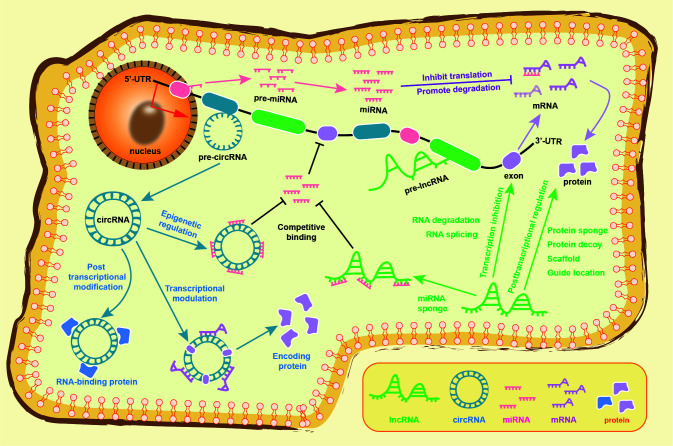
**The regulatory mechanism of the competitive endogenous RNA networks in transcription, translation, and epigenetics**. LncRNA regulates transcription by splicing and degrading RNA, while posttranscriptional regulation is controlled by decoy and sponge proteins, as well as epigenetic modification. CircRNA regulates transcription and translation by binding to miRNA and interacting with RNA-binding protein, particularly in certain circRNA can code protein. Both lncRNA and circRNA can serve as competitive endogenous RNA that bind with miRNA to regulate the translation of targeted mRNA, thereby inhibiting mRNA translation and promoting mRNA degradation. See text for details. circRNA, circular RNA; lncRNA, long non-coding RNA; miRNA, microRNA; mRNA, messenger RNA; UTR, untranslated regions.

Over the past decade, numerous competitive endogenous RNA (ceRNA) species have been discovered in eukaryotic genomes, which exhibit complex expression and regulatory mechanisms [10]. Although previous study suggested that noncoding RNAs (ncRNAs) cannot encode proteins, they participate in the regulation of many pathophysiological processes (such as SCVDs) *via* ceRNA networks [11]. NcRNAs include long non-coding RNAs (lncRNAs, lncRs), circular RNAs (circRNAs, circRs) and microRNAs (miRNAs, miRs). LncRNAs regulate transcription by splicing and degrading RNA, while posttranscriptional regulation is controlled by decoy and sponge proteins, as well as nuclear compartmentalization and epigenetic modification [12]. Different from linear RNA, circRNA consists of a covalently closed loop, which has neither a poly-A tail nor $5^{\prime }$-$3^{\prime }$ polarity. CircRNAs regulate transcription and translation by binding to miRNAs and interacting with RNA-binding proteins [13]. A novel type of epigenetic regulation known as ceRNA is considered to be a natural bait for miRNAs. CeRNA competes the miRNA response element (MRE) to modulate the expression of target messenger RNA (mRNA) [14]. Both lncRNAs and circRNAs can serve as ceRNAs that bind with miRNAs to regulate the translation of targeted mRNAs [15], which forms the lncRNA/circRNA-miRNA-mRNA axis that constitutes the basic network of ceRNAs, as shown in Fig. 2. Complex ceRNA networks play important roles in the pathogenesis of SCVDs by regulating apoptosis, immunity, endothelial dysfunction, and inflammation. Notwithstanding, the underlying mechanism of septic CVDs is not yet clear.

Increasing evidence has shown that ncRNAs are specifically expressed in some tissues and developmental stages and can be used as biomarkers for disease diagnosis and prognosis and as potential therapeutic targets [16]. The lncRNA/circRNA-miRNA-mRNA axis acts as a sophisticated interactive network that regulates cardiovascular structure and function, providing a promising breakthrough for improving cardiovascular dysfunction. This review will provide insight into sepsis induced cardiovascular toxicity and reveal directions for further research, contributing to the diagnosis, treatment, and monitoring of SCVDs.

## 2. Competing Endogenous RNAs in the Regulation of SCVDs 

### 2.1 The Roles of LncRNAs in SCVDs 

LncRNAs are a typical class of ceRNAs and are defined as transcripts with a length of more than 200 bp without obvious protein coding functions. It has been demonstrated that lncRNAs participate in multitudinous cellular functions and pathological processes by regulating epigenetic, transcriptional, and posttranscriptional levels, and are potential therapeutic targets for septic CVDs. Lipopolysaccharide (LPS) is a glycolipid heteropolymer located on the outer wall of Gram-negative bacteria, and is often used to simulate sepsis toxicity in animal and cell experiments. Zhang *et al*. [17] focused on key lncRNAs and mRNAs in septic cardiomyopathy by using an LPS-induced rat model. A total of 74 differentially expressed lncRNAs (41 downregulated and 33 upregulated, of which 39 were novel lncRNAs) and 4011 differentially expressed mRNAs (2093 downregulated and 1918 upregulated) were identified by whole genomic RNA sequencing. Subsequent analysis showed that inhibiting the upregulation of lncRNA *PVT1* (plasmacytoma variant translocation 1) significantly inhibited the expression of *Myd88* and *Bcl-2* and promoted the expression of *c-Myc* and *Bax*, thereby alleviating myocardial depression in response to LPS. Another study targeted septic vascular dysfunction by using an LPS-stimulated human umbilical vein endothelial cell (HUVEC) model [18]. A total of 30,584 differentially expressed lncRNAs (1068 downregulated and 871 upregulated) were screened by the Arraystar Human lncRNA Expression Microarray; among them, *CTC-459I6.1* and *AL132709.5* were the most downregulated and upregulated lncRNAs, respectively.

In addition, a systematic lncRNA survey was performed to assess the effects of LPS stimulation on human monocytes [19]. A total of 221 differentially expressed lncRNAs (39 downregulated and 182 upregulated) were screened in LPS-treated granulocytes, and these differentially expressed lncRNAs were associated with the human innate immune response. Notably, the LPS-induced differentially expressed lncRNAs were enriched in NF-κB binding sites, and NF-κB-dependent and subcellular transcripts (*IL1β-RBT46* and *IL1β-eRNA*) regulated the release of the key proinflammatory mediators CXCL8 and IL1β. Furthermore, another transcript survey was performed to evaluate the effects of ricin toxin (RT) stimulation on murine monocytes [20]. A total of 155 lncRNAs, 35 circRNAs, 24 miRNAs and 273 mRNAs were differentially expressed in RAW264.7 cells under RT conditions, of which 133/22 lncRNAs, 9/26 circRNAs, 11/13 miRNAs and 7/266 mRNAs were significantly downregulated/upregulated, respectively. The co-expression network of lncRNA/circRNA-miRNA-mRNA was then integrated with 2 significantly downregulated miRNAs (*mmu-miR-1930-3p* and *mmu-miR-5114*), and 10 hub genes were associated with inflammatory signaling pathways. The lncRNA-mediated ceRNA networks regulate cardiovascular toxicity in sepsis as shown in Tables 1,2 (Ref. [21, 22, 23, 24, 25, 26, 27, 28, 29, 30, 31, 32, 33, 34, 35, 36, 37, 38, 39, 40, 41, 42, 43, 44, 45, 46, 47, 48, 49, 50, 51, 52, 53, 54, 55, 56, 57, 58, 59, 60, 61]).

**Table 1.** **The lncRNA associated ceRNA networks in sepsis-induced cardiotoxicity**.

lncRNA	miRNA	Validation method	mRNA	Model	Mechanism	Ref.
*ANRIL*↑	*miR-125a*↓†	Bioinformatics prediction	N.M.	Patients with sepsis	Acts as a diagnostic biomarker for the severity and prognosis of sepsis	[21]
*CAIF*↓	*miR-16*↓	RNA pull-down	*CCL2*, *CXCL1*↑†	Sepsis‐induced chronic heart failure patients and LPS-treated AC16 cells	Inhibits CMs apoptosis and inflammation by regulating miR-16 demethylation	[22]
*CHRF*↑	*miR-221*↓	RNA pull-down	*NF-κB*↑, *JNK*	LPS-injured H9C2 cells	Promotes LPS-induced H9C2 cells injury	[23]
*CRNDE*↓	*miR-29a*↑	DLR, RNA pull-down	*SIRT1*↓	LPS-treated Wistar rats and H9C2 cells	Inhibits CMs apoptosis, ROS content, and caspase-3 activity	[24]
*CYTOR*↓	*miR-24*↑	DLR, RNA pull-down	*XIAP*↓	LPS-treated SD rats and H9C2 cells	Promotes CMs viability, inhibits CMs apoptosis and apoptosis-related protein release	[25]
*FGD5‑AS1*↓	*miR‑133a‑3p*↑	DLR, RNA pull-down	*AQP1*↓	LPS-treated HL-1 cells	Reduces inflammatory cytokines (IL-1β, IL-6, and TNF-α) expression	[26]
*GAS5*↑	*miR‑26a*↓	DLR, RNA pull-down	*HMGB1*/*NF-κB*↑	PA-stimulated H9C2 cells	Aggravates cardiac inflammatory damages	[27]
*GAS5*↑	*miR-214*↓	RNA pull-down	N.M.	Patients with sepsis, LPS-stimulated AC16 cells	Inhibits the apoptosis of CMs in sepsis	[28]
*GAS5*↑	*miR‑449*↓	DLR, RNA pull-down	*HMGB1/NF-κB*↑	CLP-induced C57BL/6 mice	Promotes myocardial depression, injury, and inflammation responses in septic mice	[29]
*H19*↓	*miR-874*↑	DLR, RIP, RNA pull-down	*AQP1*↓	LPS-treated UL-1 cells and BALB/c mice	Restores LPS dysregulated inflammatory responses and myocardial dysfunction	[30]
*H19*↓	*miR-93-5p*↑	DLR, RNA pull-down	*SORBS2*↓	LPS-treated H9C2 cells	Reverses CMs growth inhibition and mitochondrial damage	[31]
*HOTAIR*↑	*miR-1-3p*↓	DLR, RNA pull-down	*IL-6*, *TNF-α*↑	LPS-treated H9C2 cells	Inhibits LPS-induced cardiomyocyte CMs proliferation, and induces cell apoptosis and inflammation.	[32]
*KCNQ1OT1*↓	*miR-192-5p*↑	DLR, RIP, RNA pull-down	*XIAP*↓	LPS-treated SD rats and H9C2 cells	Facilitates the viability and impedes the apoptosis of CMs	[33]
*LINC00472*↓	*miR-335-3p*↑	DLR, RIP, RNA pull-down	*MAOA*↓	LPS-treated C57BL/6 mice and AC-16 cells	Suppresses LPS-induced cardiomyocyte dysfunction activated by Yin Yang-1	[34]
*LUCAT1*↑	*miR-642a*↓	DLR, RNA pull-down	*ROCK1*↑	LPS-stimulated H9C2 cells	Decreases cell viability and migration, increases cell apoptosis	[35]
*MALAT1*↑	*miR-26a*↓	DLR, RNA pull-down	*HMGB1*, *TLR4*, *NF-κB*↑	SFA-treated AC16 cells	Upregulates SFA-induced myocardial inflammatory injury	[36]
*MALAT1*↑	*miR-26a-5p*↓	DLR, RNA pull-down	*RCAN2*↑	LPS-treated H9C2 cells and SD rats	Deteriorates LPS-induced inflammation and apoptosis	[37]
*MALAT1*↑	*miR-150-5p*↓	DLR, RIP, RNA pull-down	*NF-κB*↑	LPS-treated H9C2 cells	Increases sepsis-induced cardiac inflammation	[38]
*MAPKAPK5-AS1*↑	*miR-124-3p*↓	DLR, RNA pull-down	*E2F3*↑	LPS-treated H9C2 cells and SD rats	Deteriorates LPS-induced inflammation and apoptosis	[39]
*MIAT*↑	*miR-330-5p*↓	DLR, RNA pull-down	*TRAF6*/*NF-κB*↑	LPS-treated BALB/c mice and HL-1 cells	Promotes inflammation response and oxidative stress	[40]
*MIRT2*↓	*miR-101*↑	DLR, RNA pull-down	*PI3K/AKT*↓	CLP-induced SD rats	Inhibits myocardial inflammatory response and improves cardiac structure and function	[41]
*NEAT1*↑	*miR-144-3p*↓	DLR, RIP	*NF-κB*↑	LPS-treated HL-1 cells	Suppresses CMs viability, promotes apoptosis and inflammatory response	[42]
*PTENP1*↑	*miR-106b-5p*↓	DLR, RNA pull-down	*IL-6*, *TNF-α*↑†	CLP-induced C57BL/6 mice, LPS-treated H9C2 cells	Enhances cardiac myoblast viability and attenuates inflammation via Matrine treatment	[43]
*RMRP*↓	*miR-1-5p*↑	DLR, RNA pull-down	*HSPA4*↓	LPS-treated C57B6/L mice and primary CMs	Attenuates LPS-induced CMs apoptosis and mitochondrial injury	[44]
*SNHG1*↓	*miR-181a-5p*↑	DLR, RNA pull-down	*XIAP*↓	LPS-stimulated H9C2 cells	Facilitates CMs viability and represses inflammation and oxidative stress	[45]
*TTN-AS1*↓	*miR-29a*↑	DLR, RNA pull-down	*E2F2*↓	LPS-treated SD rats and H9C2 cells	Attenuates mitochondrial ROS activity, and enhances mitochondrial membrane potential	[46]
*XIST*↑	*miR-150-5p*↓	DLR, RIP, RNA pull-down	*c-Fos*↑	CLP-induced SD rat, LPS-treated H9C2 cells	Aggravates cardiac dysfunction, increases CMs apoptosis and pyroptosis	[47]
*XIST*↑	*miR-7a-5p*↓	RNA pull-down	*PGC-1α*↓†	LPS-stimulated mouse CMs	Reduces cell apoptosis and increases cell proliferation	[48]
*ZFAS1*↓	*miR-138-5p*↑	DLR, RNA pull-down	*SESN2*↓	Patients with sepsis-induced myocardial injury, LPS-treated SD rats and H9C2 cells	Ameliorates sepsis induced CMs pyroptosis, myocardial injury and inflammatory response	[49]
*ZFAS1*↓	*miR-34b-5p*↑	DLR, RNA pull-down	*SIRT1*↓	LPS-treated SD rats and H9C2 cells	Alleviates inflammatory response and cell apoptosis	[50]
*ZFAS1*↑	*miR-590-3p*↓	DLR, RIP, RNA pull-down	*NLRP3*↑	CLP-induced C57BL/6 mice, LPS-treated primary CMs	Aggravates autophagy and pyroptosis of CMs activated by SP1	[51]

Note. ↑, upregulated in sepsis; ↓, downregulated in sepsis; †, predicted. Animals, C57BL/6 and BALB/c (mouse), SD (rat). CMs, cardiomyocytes, including HL-1, UL-1 (mouse), H9C2 (rat), AC16 (human). CLP, cecal ligation puncture; DLR, dual-luciferase reporter gene assay; LPS, lipopolysaccharide; N.M., not mentioned; PA, palmitic acid; RIP, RNA-binding protein immunoprecipitation; SFA, saturated fatty acid.

**Table 2.** **The lncRNA associated ceRNA networks of septic cardiovascular toxicity in non-cardiomyocytes**.

lncRNA	miRNA	Validation method	mRNA	Model	Mechanism	Ref.
*HULC*↑	*miR-204-5p*↓	DLR, RIP, RNA pull-down	*TRPM7*↑	LPS-stimulated HUVECs	Deteriorates cell apoptosis, inflammation, and oxidative stress	[52]
*HOTAIR*↑	*miR-211*↓	DLR, RNA pull-down	*IL-6R*↑	CLP-induced C57BL/6 mice, LPS-treated monocytes	Aggravates the progression of sepsis, inhibits cellular proliferation, and promotes monocyte apoptosis	[53]
*LUADT1*↓	*miR-195*↑	DLR, RNA pull-down	*PIM-1*↓	Sepsis patients, LPS-exposed HCAECs	Reduces LPS-induced cardiac endothelial cell apoptosis	[54]
*MALAT1*↓	*Hsa-miR-346*↑	DLR, RNA pull-down	*SMAD3*↓	Patients with sepsis, LPS-treated RAW264.7 mouse macrophages	LPS decreases *MALAT1* and *SMAD3* levels, and increases has-miR-346 level	[55]
*MALAT1*↑	*miR-23a*↓	DLR, RNA pull-down	*MCEMP1*↑	CLP-induced C57BL/6 mice, LPS-treated monocytes	Enhances the inflammation in septic mice and inhibits monocyte apoptosis	[56]
*MALAT1*↑	*miR-146*↓	DLR, RNA pull-down	*NF-κB*↑	LPS-exposed HMEC-1 cells	Promotes LPS-induced inflammatory injury in microvascular endothelial cells	[57]
*MALAT1*↑	*miR-150*↓	DLR, RIP, RNA pull-down	*NF-κB*↑	LPS-challenged HUVECs	Exacerbates endoplasmic reticulum stress and inflammatory response	[58]
*PVT1*↑	*miR-29a*↓	DLR, RNA pull-down	*HMGB1*↑	LPS-treated C57BL/6 mice and primary macrophages	Enhances macrophage M1 polarization and sepsis-induced myocardial injury	[59]
*SNHG15*↑	*miR-362-3p*↓	RNA pull-down	*TNF-α*, *IL-6*↑	LPS-exposed HUVECs	Aggravates LPS-induced vascular endothelial cell apoptosis, inflammatory factor expression and oxidative stress response	[60]
*SNHG16*↓	*miR-15a/16*↑	DLR, RNA pull-down	*TLR4*↓	LPS-stimulated RAW264.7 cells	*SNHG16* and *TLR4* were downregulated, while miR-15a and miR-16 were upregulated	[61]

Note. ↑, upregulated in sepsis; ↓, downregulated in sepsis. Animals, C57BL/6 and BALB/c (mouse), SD (rat). CLP, cecal ligation puncture; DLR, dual-luciferase reporter gene assay; HCAECs, human primary coronary artery endothelial cells; HMECs, human microvascular endothelial cells; HUVECs, human umbilical vein endothelial cells; LPS, lipopolysaccharide; RIP, RNA-binding protein immunoprecipitation.

### 2.2 The Roles of CircRNAs in SCVDs 

CircRNAs (>200 bp) are a class of covalently closed ceRNAs in the cytoplasm that lack $5^{\prime }$-$3^{\prime }$ polarity or poly-A tails, which contributes to higher stability than linear RNAs (including lncRNAs and miRNAs) [62]. CircRNAs are expected to be ideal biomarkers for disease diagnosis due to their stability and highly conserved characteristics. An increasing number of studies have screened significantly differentially expressed circRNAs in septic CVDs by high-throughput sequencing to find candidate diagnostic biomarkers. A recent study identified 11 differentially expressed circRNAs (7 up- and 4 downregulated expression) and 78 differentially expressed miRNAs (54 up- and 24 downregulated expression) in LPS-injected septic shock rat hearts, most of which were closely associated with sepsis cardiac depression [63]. Simultaneously, another study reported 801 dysregulated circRNAs (373 up- and 428 downregulated expression) and 4966 dysregulated mRNAs (2063 up- and 2903 downregulated expression) in the aortic tissue of LPS-injected septic rats, most of which were significantly enriched in the calcium signaling pathway [64]. Although high-throughput sequencing can effectively screen certain candidate genes, their regulatory mechanism and diagnostic value need to be further verified from bench to bedside.

**Fig. 3.**
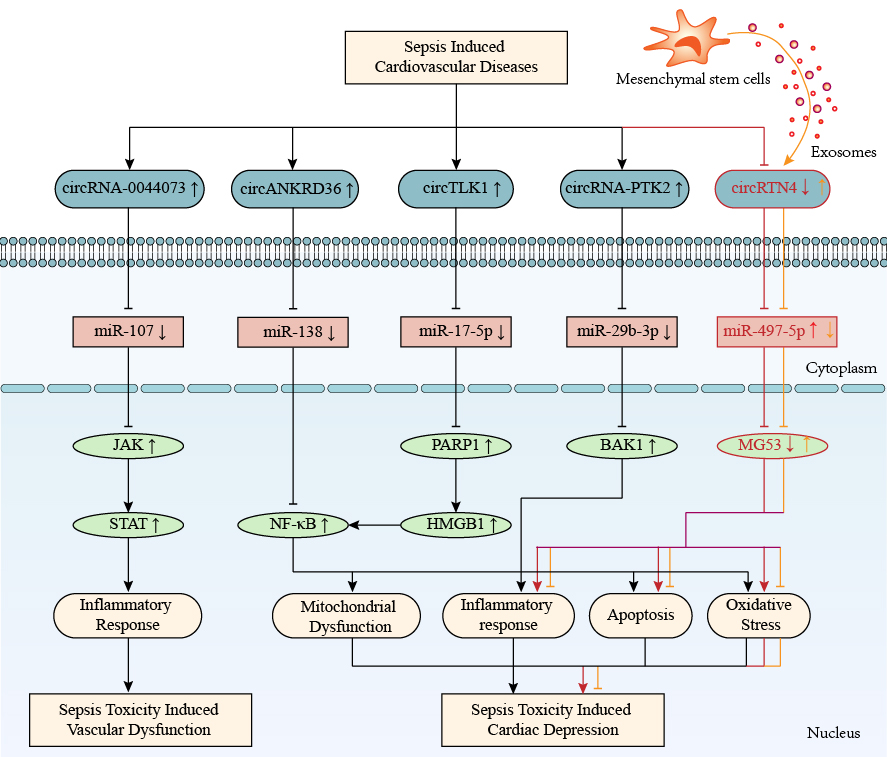
**CircRNA-mediated competitive endogenous RNA networks in sepsis-induced cardiovascular diseases**. CircRNAs *0044073, ANKRD36, TLK1, PTK2*, and *RTN4* are involved in sepsis-induced cardiovascular toxicity by regulating inflammation, mitochondrial dysfunction, oxidative stress, and apoptosis. The up-arrow indicates upregulation and the down-arrow indicates downregulation. The arrow-line represents promotion and the T-line represents inhibition. *ANKRD36*, ankyrin repeat domain 36; BAK1, BCL2-antagonist/killer 1; HMGB1, high mobility group protein 1; JAK, janus kinase; MG53, tripartite motif/TRIM72; PARP1, poly ADP-ribose polymerase 1; *PTK2*, protein tyrosine kinase 2; *RTN4*, reticulon 4; STAT, signal transducer and activator of transcription; *TLK1*, tousled-like kinase 1; circRNA, circular RNA; NF-κB, nuclear factor-κB.

The circRNA *TLK1 (circTLK1, circ_009932)* has recently been shown to be significantly upregulated in cecal ligation puncture (CLP)-induced septic rat hearts [65]. *CircTLK1* acts as a ceRNA competitor of *miR-17-5p*. The overexpression of *circTLK1* is related to downregulated *miR-17-5p* and increased levels of *PARP1* and *HMGB1*, which consequently leads to mitochondrial dysfunction and DNA oxidative damage in LPS-treated human cardiomyocytes. Similarly, circRNA *PTK2* was highly expressed in the hearts of CLP mice, and in LPS-exposed human umbilical vein endothelial cells (HUVECs) and human vascular smooth muscle cells (HUVAMCs), enhanced expression of *circRNA-0044073* promotes the proliferation of vascular endothelial and smooth muscle cells, thereby alleviating atherosclerosis [66]. *CircRNA-0044073* activates the *JAK/STAT* signaling pathway by sponging *miR-107*, as evidenced by RNA-pulldown and dual-luciferase reporter assays. Interestingly, exosomes derived from mesenchymal stem cells alleviate cardiotoxicity damage by upregulating the *circRTN4/miR-497-5p/MG53* axis, and *circRTN4* acts as a functional medium to suppress oxidative stress in CLP rats and LPS-treated H9C2 cells [67]. The circRNA-mediated ceRNA networks regulate cardiovascular toxicity in sepsis, as shown in Table 3 (Ref. [65, 66, 67, 68]) and Fig. 3.

**Table 3.** **The circRNA-mediated ceRNA networks in the regulation of cardiovascular toxicity in sepsis**.

circRNA	miRNA	Validation method	mRNA	Model	Mechanism	Ref.
*circRNA-0044073*↑	*miR-107*↓	DLR, RNA pull-down	*JAK*/*STAT*↑	LPS-stimulated HUVSMCs and HUVECs	Promotes the proliferation and invasion of HUVSMCs and HUVECs	[66]
*circTLK1*↑	*miR-17-5p*↓	DLR, RIP, RNA pull-down	*PARP1*/*HMGB1*↑	CLP-induced SD rats, LPS-treated human CMs	Aggravates mitochondrial dysfunction, DNA oxidative damage, and CMs apoptosis	[65]
*circRNA-PTK2*↑	*miR-29b-3p*↓	DLR, RNA pull-down	*BAK*1↑	CLP-induced C57BL/6 mice	Promotes inflammatory response and myocardial damage	[68]
*circRTN4*↓	*miR-497-5p*↑	DLR, RIP, RNA pull-down	*MG53*↓	CLP-induced wistar rat, LPS-treated H9C2 and AC16 cells	Mesenchymal stem cells-derived exosomes prevent sepsis-induced myocardial injury	[67]

Note. ↑, upregulated in sepsis; ↓, downregulated in sepsis. Animals, C57BL/6 (mouse) and SD (rat). CMs, cardiomyocytes, including H9C2 (rat) and AC16 (human). CLP, cecal ligation puncture; DLR, dual-luciferase reporter gene assay; HMECs, human microvascular endothelial cells; HUVSMCs, human vascular smooth muscle cells; HUVECs, human umbilical vein endothelial cells; LPS, lipopolysaccharide; RIP, RNA-binding protein immunoprecipitation; RNA-FISH, RNA fluorescent in situ hy-bridization.

### 2.3 The Roles of MiRNAs in SCVDs

MiRNAs are highly conserved and small ncRNAs with a length of approximately 21 nucleotides that can control many developmental and cellular processes in eukaryotes [69]. RNA polymerases II and III transcribe pre-miRNAs to form precursors, which then undergo complex slicing and splicing events to synthesize mature miRNAs. MiRNAs regulate biological functions through the silencing complex (RISC) induced by RNA, which activates the complex to target miRNA-specified mRNAs [70]. Recently, Liu *et al*. [71] identified 19 different expression miRNAs (5 downregulated, 14 upregulated) and 323 different expression mRNAs (11 downregulated, 312 upregulated) in RAW264.7 cells under RT conditions, and 713 miRNA-mRNA networks were enrolled in the T-cell receptor, *MAPK*, and *JAK-STAT* signaling pathways. TargetScan prediction and validation experiments revealed that *miR-155-3p* could bind to *GAB2* and reduce TNF-α secretion. Additionally, *curcumin* (a natural polyphenolic compound) downregulated *miR-155* levels in LPS-treated RAW264.7 and THP-1 cells, and promoted macrophage survival and inhibited inflammatory cytokine release (TNF-α and IL-6) [72].

Extensive knowledge suggests that miRNA activation or repression plays a vital role in the regulation of septic CVDs by interacting with lncRNAs, circRNAs and their target mRNAs. *MiR-145 * [73] and *miR-29a * [74] were downregulated in LPS-stimulated H9C2 cells, while *geniposide* (an iridoid glycoside of *Gardenia jasminoides Ellis*) and *gracillin* (a steroidal saponin of *Dioscorea quinqueloba*) impeded apoptosis and inflammation by upregulating *miR-145* and *miR-29a* levels, and blocking the *MEK/ERK* and *NF-κB* signaling pathways, respectively. Besides, *miR-223 * [75] and *miR-429 * [76] were upregulated in LPS-treated H9C2 cells, while *emodin* (a natural product from *Rheum palmatum*) and *swainsonine* (a natural alkaloid from *Locoweed*) protected cardiomyocytes against LPS-caused apoptosis and inflammatory damage through downregulating *miR-223* and *miR-429*, and activating the *JNK* and *p38-MAPK/NF-κB* signaling pathways, respectively. Furthermore, *miR-513a-5p* was upregulated in HUVECs treated with TNF-α and LPS, whereas negative regulation of *miR-513a-5p* could alleviate endothelial cell apoptosis by promoting the expression of X-linked inhibitor of apoptosis (*XIAP*) [77].

## 3. Competing Endogenous RNA Networks in SCVDs 

### 3.1 Inflammation 

**Fig. 4.**
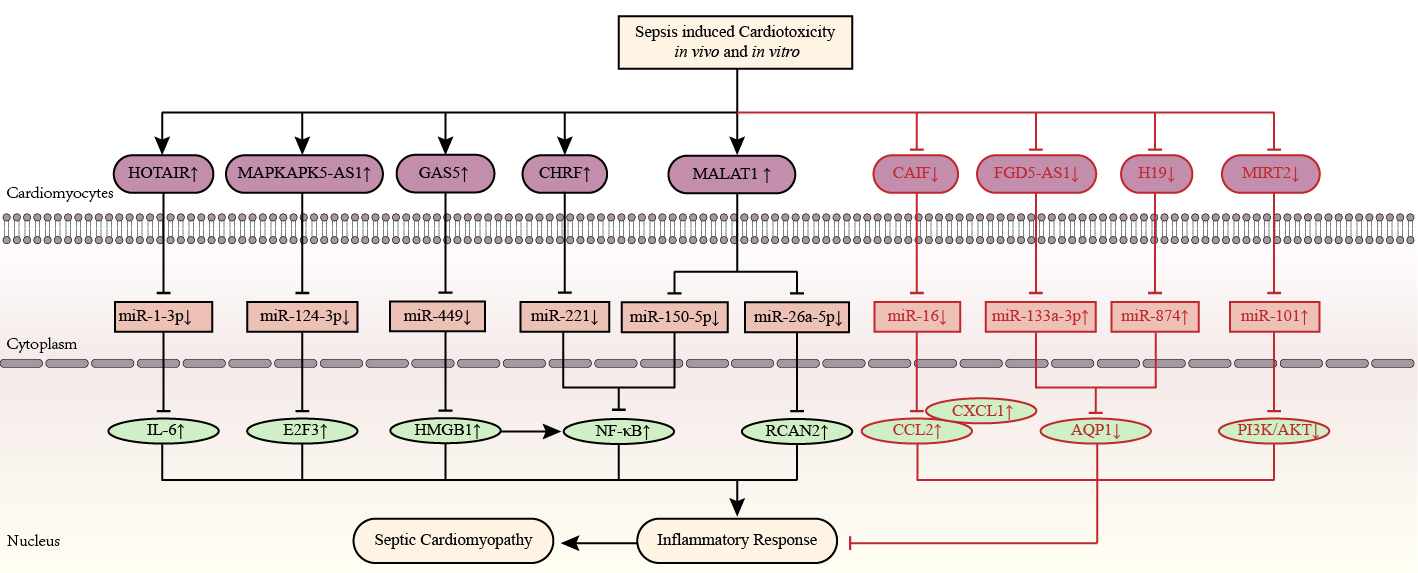
**Inflammation-related competitive endogenous RNA networks in sepsis toxicity-induced cardiomyopathy**. LncRNAs *HOTAIR, MAPKAPK5-AS1, GAS5, CHRF*, and *MALAT1* are upregulated, while lncRNAs *CAIF, FGD5‑AS1, H19*, and *MIRT2* are downregulated in septic cardiomyopathy. The up-arrow indicates upregulation and the down-arrow indicates downregulation. The arrow-line represents promotion and the T-line represents inhibition. AQP1, aquaporin protein 1; *CAIF*, cardiac autophagy inhibitory factor; CCL2, chemokine ligand 2; *CHRF*, cyocardial hypertrophy related factors; CXCL1, chemokine (CXC motif) ligand 1; E2F3, E2F transcription factor 3; *FGD5‑AS1*, FGD5 antisense RNA 1; *GAS5*, growth arrest specific 5; *H19*, H19 imprinted maternally expressed transcript; HMGB1, high mobility group protein 1; *HOTAIR*, HOX transcript antisense RNA; IL, interleukin; *MALAT1*, metastasis associated lung adenocarcinoma transcript 1; *MAPKAPK5-AS1*, MAPKAPK5 antisense RNA 1; MIRT2, myocardial infarction-related transcription factors 2; NF-κB, nuclear factor-κB; PI3K/Akt, phosphatidylinositol-3-kinase/protein kinase B; RCAN2, regulator of calcineurin 2.

The typical clinical phenotypes of sepsis are systemic inflammatory response syndrome (SIRS), which results in fever, tachypnea, tachycardia, and peripheral leukocytosis and is a dysregulated host response to infection that leads to a hyperinflammatory cytokine storm and even immunodepression [78]. Sepsis consists of two stages, acute immune activation and chronic immune depression. During the initial activation phase of sepsis, necrotic tissue and microorganisms produce damage-associated molecular patterns (DAMPs) and pathogen-associated molecular patterns (PAMPs), which then activate pattern recognition receptors (PRRs, such as TLRs) on the cytomembrane, triggering a series of intracellular signal events and leading to a cascade of release of inflammatory factors (such as NF-κB, IL-6, TNF-α, HMGB1, and NLRP3). Mounting evidence suggests that ncRNAs are involved in the initiation and progression of sepsis-activated inflammation, as shown in Fig. 4.

The lncRNA ribonucleic acid nuclear paraspeckle assembly transcript 1 (*NEAT1*) was significantly increased in LPS-treated C57 mice and HL-1 mouse cardiomyocytes [42, 79]. Silencing *NEAT1* inhibited inflammation-mediated cardiomyocyte apoptosis, and this protective effect was similar to that of *miR-144-3p* overexpression [42]. Both* in vivo* and* in vitro* experiments indicated that NEAT1 was an upstream regulator of *NF-κB* and a ceRNA of *miR-144-3p*. The lncRNA MAP kinase-activated protein kinase 5 antisense gene protein 1 (*MAPKAPK5-AS1*) was significantly upregulated in LPS-treated SD rats and H9C2 cells [39]. Knockdown of *MAPKAPK5-AS1* inhibited inflammatory response by targeting *miR-124-3p* to downregulate the expression of E2F3. Besides, the lncRNA cardiac hypertrophy related factor (*CHRF*) was significantly upregulated in LPS-treated H9C2 cells [23]. Knockdown of *CHRF* by small interfering RNAs (siRNAs) attenuated LPS-induced cardiomyocyte apoptosis and the release of inflammatory factors (TNF-α and IL-6). Silencing CHRF suppressed the activation of the NF-κB and JNK pathways, and this effect could be partially blocked by co-transfection with a *miR-221* inhibitor. These results illustrate that the *NEAT1/miR-144-3p/NF-κB* and *CHRF/miR-221/NF-κB/JNK* axes may represent potential pathways by which cardiomyocytes resist septic inflammatory injury.

Abnormal circulating ncRNAs can be used as biomarkers to evaluate the risk, severity, and prognosis of sepsis. Gui *et al*. [21] found that the plasma lncRNA antisense ncRNA in the *INK4* locus (*ANRIL*) was significantly elevated in sepsis patients (aged 56.6 ± 13.0 years) compared with healthy controls and was accompanied by decreased miR-125a levels. A high plasma *ANRIL/miR-125a* ratio was an independent predictor of decreased cumulative survival and increased 28-day mortality. Another study showed that plasma lncRNA *H19* was significantly decreased in sepsis patients (aged 71.3 ± 9.7 years), which was accompanied by elevated miR-874 levels [30]. Positively regulating *H19* or negatively regulating miR-874 blunts LPS-mediated cardiomyocyte apoptosis. Aquaporin protein 1 (*AQP1*) acts as the target of *miR-874* and is regulated by *H19*. Moreover, *AQP1* can be regulated by the lncRNA *FGD5-AS1/miR-133a-3p* axis in LPS-treated HL-1 cells, protecting cardiomyocytes from septic injury [26]. The upregulation of *FGD5-AS1* increases the expression of *AQP1* by downregulating *miR-133a-3p* expression.

### 3.2 Oxidative Response

**Fig. 5.**
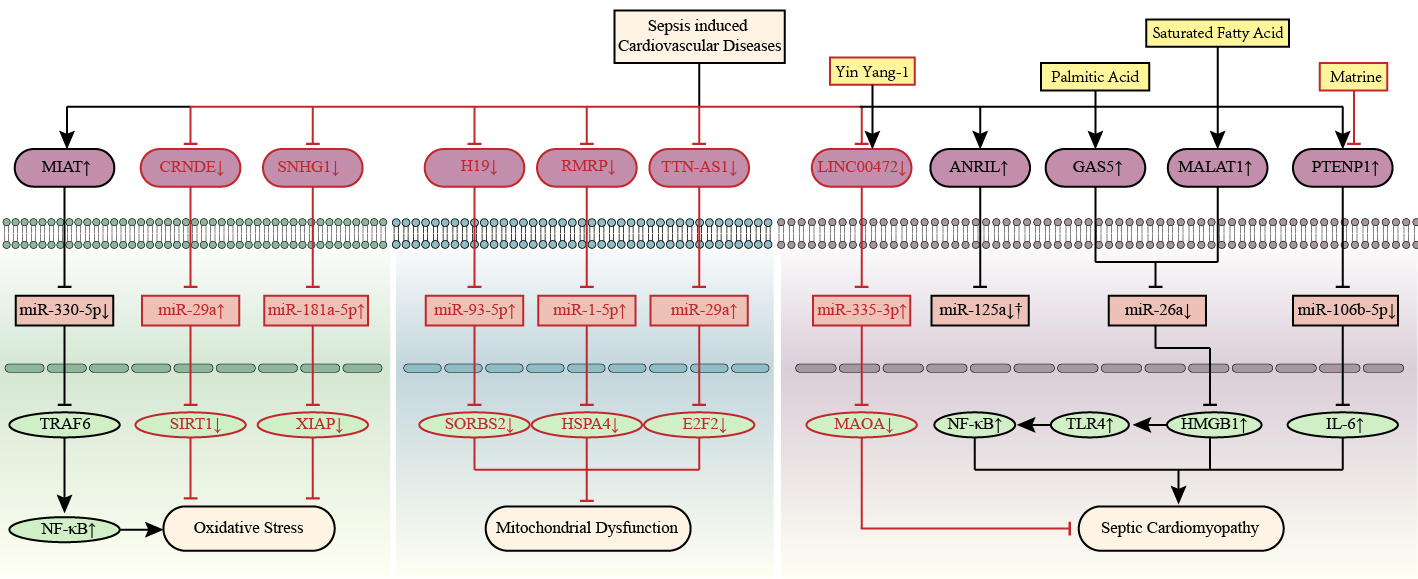
**LncRNA-mediated competitive endogenous RNA networks in septic cardiomyopathy**. LncRNAs *CRNDE, MIAT*, and *SNHG1* are associated with septic cardiomyopathy by regulating oxidative stress. LncRNAs *H19, RMRP*, and *TTN-AS1* are associated with septic cardiomyopathy by regulating mitochondrial injury. Yin Yang-1 and matrine alleviate septic cardiomyopathy by upregulating *LINC00472* and downregulating *PTENP1*, respectively. Palmitic acid and saturated fatty acid aggravate septic cardiomyopathy by upregulating *GAS5* and *MALAT1*, respectively. The up-arrow indicates upregulation and the down-arrow indicates downregulation. The arrow-line represents promotion and the T-line represents inhibition. ANRIL, antisense ncRNA in the INK4 locus; *CRNDE*, colorectal neoplasia differentially expressed; E2F2, E2F transcription factor 2; *GAS5*, growth arrest specific 5; *H19*, H19 imprinted maternally expressed transcript; HMGB1, high mobility group protein 1; HSPA4, heat shock 70 kDa protein 4; IL-6, interleukin-6; *MALAT1*, metastasis associated lung adenocarcinoma transcript 1; MAOA, monoamine oxidase A; MIAT, myocardial infarction associated transcript; NF-κB, nuclear factor-κB; *PTENP1*, PTEN pseudogene-1; *RMRP*, RNA component of mitochondrial RNA processing endoribonuclease; SIRT1, sirtuins 1; *SNHG16*, small nucleolar RNA host gene 16; SORBS2, sorbin and SH3 domain‐containing 2; TLR4, toll-like receptor 4; TRAF6, TNF receptor associated factor; *TTN-AS1*, TTN antisense RNA 1; XIAP, X-linked inhibitor of apoptosis gene; lncRNA, long non-coding RNA.

Along with inflammation, oxidative stress-mediated injury participates in detrimental pathways activated during sepsis-related organ dysfunction, ultimately causing multiple organ failure and death. Sepsis accelerates the excessive production of reactive oxygen species (ROS) and the disruption of antioxidant systems, disequilibrating redox homeostasis to a prooxidative state. Once the pathogen-caused prooxidant state is established, the subsequent cascade release of ROS exacerbates further self-damage to injured cells, independent of the original pathogen itself. Excessive ROS levels directly cause cardiomyocyte apoptosis, mitochondrial damage and cardiac insufficiency in the septic myocardium, simultaneously, it leds to glycocalyx degradation, increased permeability and impaired vasoreactivity in the septic vascular endothelium [80]. Accumulating knowledge implicates that ceRNA networks are involved in regulating the pathogenic process of sepsis-related CVDs, as shown in Fig. 5.

LncRNA myocardial infarction associated transcript (*MIAT*) is overexpressed in LPS-treated BALB/c mice and HL-1 cells [40]. Transfection of *MIAT* siRNA antagonized LPS-induced oxidative responses in HL-1 cells, whereas *miR-330-5p* mimics partially reversed these effects, as evaluated by ROS and malondialdehyde (MDA) assays, mitochondrial membrane potential (MMP), and the glutathione reduction/oxidation (GSH/GSSG) ratio. MIAT regulates *miR-330-5p* directly as an endogenous sponge and activates the *TRAF6/NF-κB* axis by targeting *miR-330-5p*, which is accompanied by the overexpression of TNF-α, IL-1β and IL-6. In contrast, the lncRNA *MIRT2* improves cardiac structure and function in CLP-treated rats by modulating the *miR-101/PI3K/AKT* axis [41]. Moreover, the lncRNA colorectal neoplasia differentially expressed (*CRNDE*) and small nucleolar RNA host gene 1 (*SNHG1*) are downregulated in LPS-induced H9C2 cells [24, 45]. The upregulation of *CRNDE* and *SNHG1* decreased ROS and MDA levels and increased superoxide dismutase (SOD) levels in H9C2 cells under LPS conditions, and these beneficial effects were partially abolished by the upregulation of *miR-29a* and *miR-181a-5p*, respectively. Dual-luciferase reporter and RNA pulldown assays demonstrated that *CRNDE* modulates *SIRT1* by sponging miR-29a and that *SNHG1* modulates *XIAP* by sponging miR-181a-5p. Hence, the *CRNDE/miR-29a/SIRT1* and *SNHG1-miR-181a-5p-XIAP* networks provide potential targets in the oxidative stress damage associated with septic cardiovascular dysfunction.

### 3.3 Endothelial Dysfunction 

The vascular endothelium acts as an important biological barrier of the circulatory system that controls systemic fluid regulation and plays key roles in hemodynamics, circulatory immunity, and tissue metabolism [81]. Endothelial dysfunction occurs in the early stage of sepsis toxic injury, which triggers the circulatory system and causes insufficient blood supply to vital organs, followed by the collapse of the immune system leading to systemic inflammation [82]. Sepsis-induced endothelial dysfunction increases the risk of CVDs, but the specific molecular mechanism is not clear. Singh *et al*. [18] examined the expression of lncRNAs and mRNAs in HUVECs stimulated with LPS, and 30,584 lncRNAs (1068 downregulated and 871 upregulated) and 26,106 mRNAs (536 downregulated and 733 upregulated) were significantly differentially expressed; among them, *CTC-459I6.1* was the most downregulated, and *AL132709.5* was the most upregulated lncRNA, which are associated with sepsis-induced endothelial dysfunction.

**Fig. 6.**
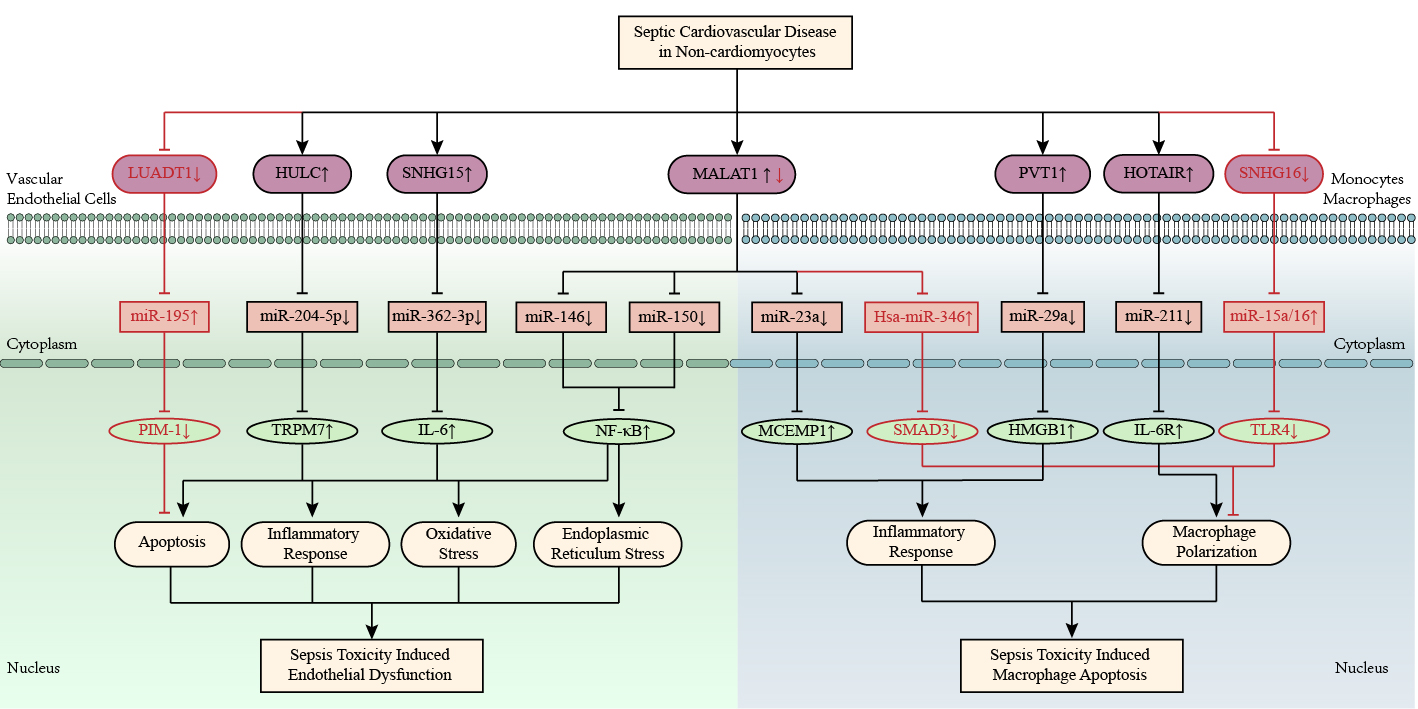
**LncRNA-mediated ceRNA networks in vascular endothelial cells and mononuclear macrophages under sepsis environment**. LncRNAs *LUADT1, HULC*, and *SNHG15* are involved in sepsis endothelial dysfunction. LncRNAs *PVT1, HOTAR*, and *SNHG16* are involved in sepsis-induced macrophage polarization. In general, lncRNA *MALAT1* aggravates sepsis-induced endothelial dysfunction and macrophage polarization, but *MALAT1* inhibits LPS-induced RAW264.7 macrophage apoptosis by regulating has-miR-346/SMAD3 axis. The up-arrow indicates upregulation and the down-arrow indicates downregulation. The arrow-line represents promotion and the T-line represents inhibition. HMGB1, high mobility group protein 1; *HOTAIR*, hox transcript antisense RNA; HULC, highly upregulated in liver cancer; IL-6, interleukin-6; LUADT1, lung adenocarcinoma transcript 1; *MALAT1*, metastasis associated lung adenocarcinoma transcript 1; MCEMP1, mast cell expressed membrane protein 1; NF-κB, nuclear factor-κB; PIM-1, pim-1 proto-oncogene; *PVT1*, plasmacytoma variant translocation 1; SMAD3, SMAD family member 3; *SNHG15/16*, small nucleolar RNA host gene 15/16; TLR4, toll-like receptor 4; TRPM7, transient receptor potential melastatin-subfamily member 7; lncRNA, long non-coding RNA.

A variety of lncRNAs have been shown to participate in septic endothelial dysfunction, such as *MALAT1* (metastasis-associated lung adenocarcinoma transcript 1), *LUADT1* (lung adenocarcinoma transcript 1), and *HULC* (highly upregulated in liver cancer), as shown in Fig. 6. *MALAT1* regulates *miR-146* to inhibit the activation of NF-κB and protects human microvascular endothelial cells (HMEC-1) from inflammatory damage induced by LPS [57]. *LUADT1* promotes *PIM-1* expression by sponging *miR-195* to protect against apoptosis in LPS-exposed human primary coronary artery endothelial cells (HCAECs) [54]. *HULC* deteriorates cell apoptosis, inflammatory reaction, and oxidative stress through *miR-204-5p/TRPM7* in LPS-stimulated HUVECs [52]. Mechanistically, *MALAT1/miR-146/NF-κB*, *LUADT1/miR-195/PIM-1*, and *HULC/miR-204-5p/TRPM7* are critical signaling pathways that regulate LPS-induced endothelial injury and associated vascular paralysis. These findings reveal novel targets in the lncRNA-miRNA-mRNA axis associated with endothelial dysfunction and provide potential biomarkers for sepsis-related vascular diseases.

### 3.4 Macrophage Polarization 

Macrophages are involved in the regulation of innate and acquired immunity and can be transformed into classically (M1) and alternatively (M2) activated macrophages under different pathophysiological conditions. M1/M2 polarization of macrophages is positively correlated with the severity of sepsis, M1-type macrophages release proinflammatory factors to participate in the occurrence and maintenance of sepsis, and M2-type macrophages release anti-inflammatory factors to participate in the resolution of sepsis [83]. Downregulation of lncRNA *PVT1* suppresses M1-type macrophage polarization [59]. *PVT1* inhibited *miR-29a* expression and upregulated HMGB1 expression, subsequently aggravating sepsis-caused myocardial injury in LPS-treated C57BL/6 mice and primary macrophages. A recent study reported that *MALAT1* was downregulated in patients with sepsis and LPS-induced RAW264.7 murine macrophages, while *hsa-miR-346* was upregulated [55]. *MALAT1* promoted the expression of SMAD3 by downregulating *hsa-miR-346* levels. The viability of RAW264.7 cells was inhibited by *MALAT1* and promoted by *hsa-miR-346*. Moreover, knockout of *NEAT1* significantly decreased the levels of IL-1β, IL-6, TNF-α and COX-2 in ox-LDL-stimulated THP-1 human macrophages, and the *NEAT1/miR-342-3p/NFIA* axis may be enrolled in the inflammatory response and lipid uptake in atherosclerosis [84].

In contrast, plasma lncRNA *SNHG16* (small nucleolar RNA host gene 16) expression was significantly elevated in atherosclerosis patients [85], but significantly decreased in neonatal sepsis patients [61]. *SNHG16* was significantly elevated in THP-1 cells in composite medium (containing ox-LDL, IL-1β, IL-6, IL-8, and TNF-α) [85], whereas it was significantly decreased in LPS-stimulated RAW264.7 cells [61]. Both the *SNHG16/miR-17-5p/NF-κB* and *SNHG16-miR-15a/16-TLR4* axes are involved in sepsis-induced macrophage polarization, as shown in Fig. 6 [61, 85]. We hypothesize that the opposing effects may be related to different mechanisms, pathogenesis, and disease severity. Another hypothesis is that *SNHG16* has distinct subtypes, as different types of macrophages exert opposite effects on the progression of sepsis. For example, the lncRNAs *SNHG1* and *SNHG15* are expressed at low and high levels in LPS-stimulated H9C2 cells and HUVECs, respectively [45, 60]. SNHG1 attenuates the LPS-mediated inflammatory response in cardiomyocytes by targeting *miR-181a-5p* [45], whereas *SNHG15* aggravates sepsis-associated inflammation in vascular endothelial cells by targeting *miR-362-3p * [60].

### 3.5 Mitochondrial Dysfunction 

Mitochondria are not only the core organelles that produce ROS, but also the energy metabolism factories that produce adenosine triphosphate (ATP). Sepsis promotes the production of ROS and inhibits the production of ATP in myocardial mitochondria, resulting in mitochondrial biogenesis (apoptosis and mitophagy), oxidative stress, calcium overload, energy imbalance, and cardiac dysfunction. In recent years, the epigenetic regulatory mechanisms in sepsis-related CVDs, including ncRNA regulation, chromatin remodeling, DNA methylation, and histone modifications, have attracted great attention from the life science community [86]. Evidence-based studies indicate that ceRNA networks play crucial roles in biological activities, especially in the regulation of mitochondrial function in septic CVDs, as shown in Fig. 5.

Recently, Shi and colleagues [87] identified 1275 dysregulated lncRNAs and 2769 dysregulated mRNAs in septic mouse hearts, among which 11 differentially expressed mitochondria-related mRNAs were highly correlated with 14 lncRNAs. According to recent reports, the lncRNAs *H19*, *RMRP* (RNA component of mitochondrial RNA processing endoribonuclease), and *TTN-AS1* (TTN antisense RNA 1) were downregulated in cardiomyocytes treated with LPS, whereas *miR-93-5p*, *miR-1-5p*, and *miR-29a* were upregulated [31, 44, 46]. Overexpression of *H19* alleviated mitochondrial damage and reversed cardiomyocyte growth inhibition and apoptosis *via* the *miR-93-5p/SORBS2* axis [31]. *RMRP* increased the MMP in LPS-induced primary cardiomyocytes from male C57BL/6 mice by regulating the *miR-1-5p/HSP70* axis, thereby preventing mitochondrial dysfunction and cardiomyocyte apoptosis [44]. *TTN-AS1* inhibited the sepsis-induced reduction of the MMP in H9C2 cells by regulating the *miR-29a/E2F2* pathway and suppressed inflammatory cytokine release and ROS activity [46]. Thus, *H19/miR-93-5p/SORBS2*, *RMRP/miR-1-5p/HSP70*, and *TTN-AS1/miR-29a/E2F2* perform pivotal roles in the modulation of LPS-induced mitochondrial dysfunction and related metabolic disorders.

### 3.6 Endoplasmic Reticulum Stress

In response to misfolded proteins in the endoplasmic reticulum (ER) and dysregulation of calcium homeostasis, ER stress (ERS) acts as a protective stress response by reducing intracellular unfolded proteins to prevent their aggregation [88]. Activating transcription factor 6 (ATF6), pancreatic endoplasmic reticulum kinase (PERK), and inositol-requiring enzyme 1α (IRE1α) signaling are major ERS-related pathways involved in the ER overload response, unfolded protein response, and caspase-12-mediated apoptosis. Mitochondria-associated membranes (MAMs) located on the ER are the essential sites contacting mitochondria to maintain mitochondrial function and mediate bidirectional communications, including mitochondrial DNA synthesis and fission, lipid biosynthesis, and calcium exchange [89].

The lncRNA discrimination antagonizing ncRNA (*DANCR*) is recognized as a protective factor for myocardial infarction, and a recent study revealed its mechanism in ERS-induced myocardial injury [90]. The upregulation of *DANCR* promotes autophagy and inhibits apoptosis to protect cardiomyocytes against tunicamycin-caused ERS injury. Mechanistically, *DANCR* enhances autophagy and ERS to maintain cellular homeostasis, leading to a reduction in apoptosis by adsorbing *miR-6324*. In addition, lncRNA *MALAT1* affects ERS in CLP-induced septic mice and LPS-challenged HUVECs *via* the *miR-150/NF-κB* pathway, leading to endothelial damage [58]. Downregulation of *MALAT1* inhibited the expression of the ERS-associated proteins *GRP78* and *CHOP*, along with the apoptosis-associated proteins *caspase-3* and *Bax-1*, and these effects could be blocked by a *miR-150* antagonist through regulation of *NF-κB*. These data suggest that the *MALAT1/miR-150/NF-κB* axis may contribute to LPS-induced ERS in septic CVDs.

### 3.7 Apoptosis 

**Fig. 7.**
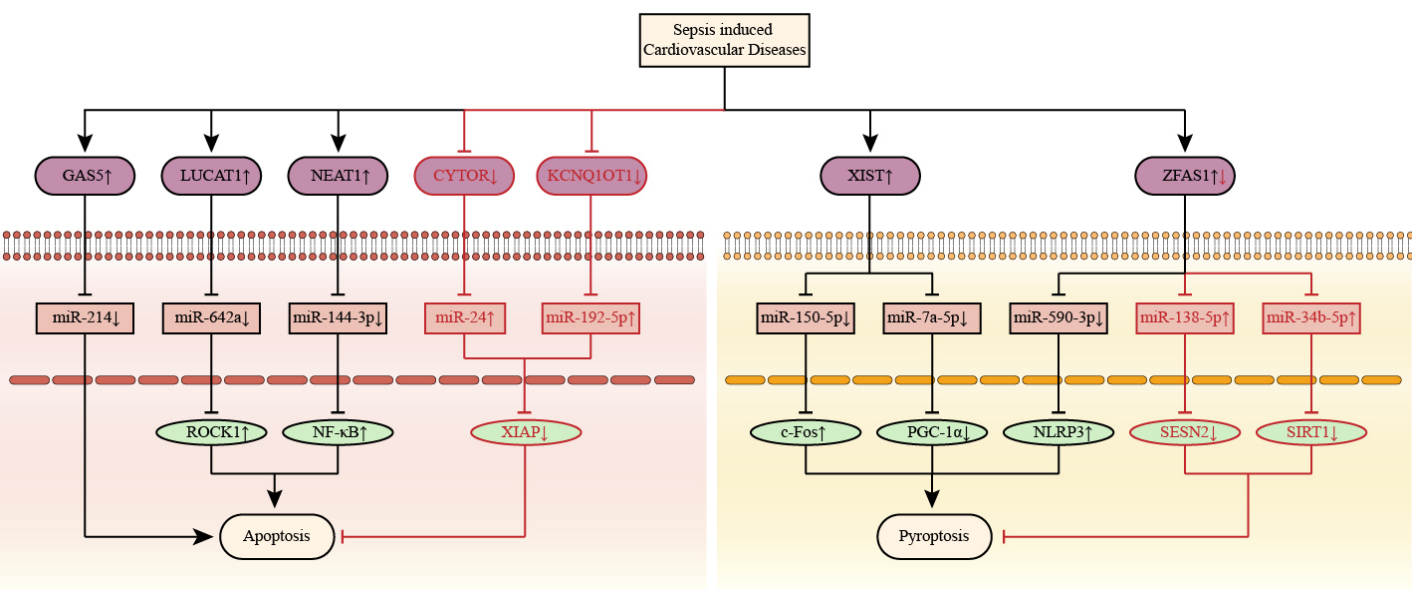
**Apoptosis and pyroptosis associated competitive endogenous RNA networks in septic cardiomyopathy**. LncRNAs *CYTOR, GAS5, KCNQ1OT1, LUCAT1*, and *NEAT1* are associated with septic cardiomyopathy by regulating apoptosis. LncRNAs *XIST* and *ZFAS1* are associated with septic cardiomyopathy by regulating pyroptosis. The up-arrow indicates upregulation and the down-arrow indicates downregulation. The arrow-line represents promotion and the T-line represents inhibition. c-Fos, A nuclear phosphoprotein; *CYTOR*, cytoskeleton regulator RNA; *GAS5*, growth arrest specific 5; *KCNQ1OT1*, KCNQ1 opposite strand/antisense transcript 1; *LUCAT1*, lung cancer-related transcript 1; *NEAT1*, nuclear paraspeckle assembly transcript 1; NF-κB, nuclear factor-κB; NLRP3, NOD-like receptor thermal protein domain associated protein 3; PGC-1α, peroxisome proliIerators-activated receptor γ coactivator l alpha; ROCK1, rho associated coiled-coil containing protein kinase 1; SESN2, sestrin 2; SIRT1, silent information regulator 1; XIAP, X-linked inhibitor of apoptosis gene; *XIST*, X-inactive specific transcript; *ZFAS1*, zinc finger antisense 1; lncRNA, long non-coding RNA.

Apoptosis refers to autonomous programmed cell death, which is strictly controlled by genes to maintain cellular homeostasis. Sepsis promotes an uncontrolled inflammatory cascade and immunocyte apoptosis that leads to immune paralysis. Targeted regulation of apoptosis can improve the survival of patients with sepsis [91]. Excessive apoptosis of immune cells induces immunosuppression, and apoptotic cardiovascular cells have the potential to exacerbate secondary heart failure and microvascular dysfunction during sepsis. Although apoptosis is a crucial event in the pathology of sepsis-induced CVDs, the underlying mechanisms are not fully understood. Increasing knowledge indicates that ncRNAs are involved in the regulation of apoptosis in sepsis-related CVDs, as shown in Fig. 7.

In LPS-stimulated H9C2 cells and septic patients, the level of *miR-642a* was significantly decreased [35]. Silencing of the lncRNA lung cancer-related transcript 1 (*LUCAT1*) attenuated LPS-induced cardiomyocyte apoptosis, and *LUCAT1* could regulate the secretion of *ROCK1* by interacting with *miR-642a*. *LUCAT1* could function as a sponge for *miR-642a* to modulate the expression of ROCK1 in LPS-exposed H9C2 cells. Beyond that, the plasma levels of the lncRNA cardiac autophagy inhibitory factor (*CAIF*) and *miR-16* were decreased in patients with chronic heart failure (CHF) caused by sepsis [22]. The overexpression of *CAIF* or *miR-16* repressed LPS-caused cardiomyocyte apoptosis by enhancing *Bcl-2* levels and reducing *Bax* levels, and the expression of *IL-6*, *CXCL1*, and *CCL2* was downregulated. *CAIF* upregulation inhibited cardiomyocyte inflammation and apoptosis by demethylating *miR-16* in sepsis-associated CHF. In addition, the lncRNA hox transcript antisense RNA (*HOTAIR*) was observed to be upregulated in LPS-treated H9C2 cells and monocytes, which targets *miR-1-3p* and *miR-211* to regulate IL-6 and IL-6R, respectively [32, 53]. These results showed that the *HOTAIR-miR-1-3p/ miR-211-IL-6* pathway was involved in sepsis-related cardiovascular toxicity.

Interestingly, some transcription factors and herbal extracts, such as *Yin Yang 1* (YY1) [34] and *matrine * [43], can inhibit sepsis-induced cardiovascular toxicity and cardiomyocyte apoptosis by regulating lncRNA-miRNA-mRNA networks. YY1 promoted the expression of lncRNA *LINC00472* in LPS-exposed AC-16 cardiomyocytes [34]. Knockdown of *LINC00472* reversed the inflammatory response and cardiomyocyte apoptosis caused by LPS and YY1 treatment, whereas the downregulation of *miR-335-3p* and upregulation of MAOA partly abrogated the protective effects mediated by *LINC00472* knockdown. Thus, YY1 contributed to SCVD progression by activating the *LINC00472/miR-335-3p/MAOA* pathway. Besides, *matrine* inhibited the expression of the lncRNA *PTENP1* (PTEN pseudogene-1) and promoted the expression of *miR-106b-5p* in CLP-induced mice and LPS-treated H9C2 cells [43]. *PTENP1* upregulation or *miR-106b-5p* downregulation reversed the protective effects of *matrine*, and *miR-106b-5p* overexpression abolished the protective effects of *PTENP1*. It seemed that *matrine-*mediated *PTENP1* deactivation protected cardiomyocytes from sepsis-induced apoptosis by targeting *miR-106b-5p*.

### 3.8 Pyroptosis 

Pyroptosis has been identified as a specialized programmed cell death caused by an uncontrolled cascade of inflammatory factors and is one of the important mechanisms involved in septic CVDs. In contrast to apoptosis-induced nuclear destruction, a typical feature of pyroptosis is that the nucleus usually remains intact. Inflammasomes (such as NLRP3, also known as NACHT, LRR, and PYD domain-containing protein) and LPS can activate certain proteins in the caspase family (caspase-1, -4, -5, and -11) to proteolytically cleave N-terminal of Gasdermin D (GSDMD) [78]. IL-18 and IL-1β can be activated by caspase-1, thereby aggravating inflammatory injury. Cleaved GSDMD is translocated to the membrane to form pores, resulting in cell swelling, membrane blebbing, DNA fragmentation and eventual cell disassembly. Wang *et al*. [47] studied the role of lncRNA X-inactive specific transcript (*XIST*) in the modulation of sepsis-caused myocardial pyroptosis. *XIST* expression was upregulated and *miR-150-5p* expression was downregulated in CLP-induced SD rat hearts and LPS-exposed H9C2 cardiomyocytes. Silencing XIST inhibited the LPS-mediated upregulation of pyroptotic proteins (NLRP3, cleaved caspase-1, and ASC) in H9C2 cells by regulating *miR-150-5p* and inhibiting c-Fos expression. Knockdown of *XIST* reduced pyroptosis-caused cardiac dysfunction in septic rats. c-Fos coupled with the promoter of the thioredoxin-interacting protein (TXNIP) gene and then promoted the expression of TXNIP. Another study showed that *XIST* bound to *miR-7a-5p* downregulated *PGC-1α* and *TFAM* expression, thereby improving LPS-stimulated mouse cardiomyocyte apoptosis and inflammatory cytokine release [48]. Taken together, these data suggest that *XIST* affects pyroptosis-mediated septic cardiovascular injury by regulating the *miR-150-5p/c-Fos/TXNIP* and *miR-7a-5p/PGC-1α/TFAM* axes, as shown in Fig. 7.

Acting as a circulating biomarker for sepsis, high plasma levels of the lncRNA zinc finger antisense 1 (*ZFAS1*) are negatively associated with disease risk, inflammatory levels, and long-term mortality [92]. *ZFAS1* has been reported to be involved in sepsis-induced multiple organ dysfunction, including acute injury of the heart, lung, and kidneys. In LPS-induced SD rats and H9C2 cardiomyocytes, the overexpression of *ZFAS1* ameliorated sepsis-induced cardiomyocyte pyroptosis and myocardial damage by targeting the *miR-138-5p/SESN2* and *miR-34b-5p/SIRT* axes [49, 50]. Both *miR-138-5p* and *miR-34b-5p* are ceRNAs of *ZFAS1*, while *miR-138-5p* and *miR-34b-5p* can negatively regulate *SESN2* and *SIRT*, respectively, indicating that the *ZFAS1/miR-138-5p/SESN2* and *ZFAS1/miR-34b-5p/SIRT* axes play critical roles in sepsis-mediated pyroptosis. Furthermore, SP1 (a zinc finger transcription factor) upregulated *ZFAS1* expression in LPS-induced mouse neonatal cardiomyocytes and inhibited cardiomyocyte pyroptosis by regulating Notch signaling [93]. In contrast, another study showed completely opposite results for SP1- and *ZFAS1*-mediated regulation of septic cardiac dysfunction [51]. *ZFAS1* was highly expressed in the septic myocardiopathy model *in vivo* and *in vitro*. SP1-activated *ZFAS1* exacerbated cardiomyocyte pyroptosis by regulating *miR-590-3p/AMPK/mTOR* signaling. Knockdown of *ZFAS1* alleviated LPS-induced pyroptosis. In conclusion, *ZFAS1* acts as a double-edged sword in the regulation of pyroptosis-caused septic CVDs through the ceRNA-miRNA-mRNA axis.

## 4. Conclusions 

**Fig. 8.**
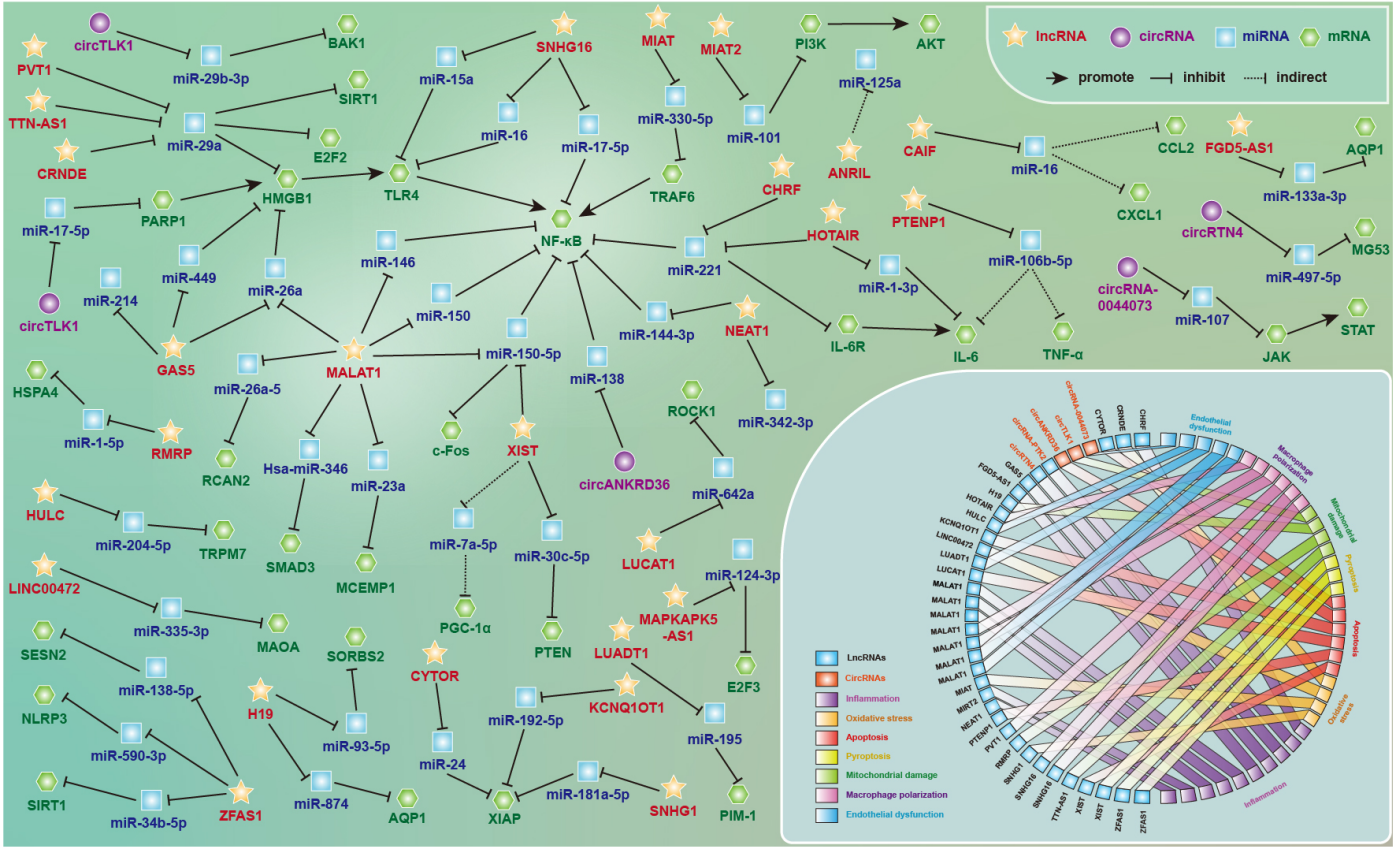
**The lncRNA- and circRNA-associated competitive endogenous RNA networks in septic cardiovascular dysfunction**. The regulatory mechanisms and potential functions of ceRNA networks in septic cardiomyopathy and vascular paralysis by regulating inflammation, oxidative response, endothelial dysfunction, macrophage polarization, apoptosis and pyroptosis, along with metabolic energy impairment and endoplasmic reticulum stress. See text for details.

Accumulating evidence has shown that ceRNA networks play vital roles in the pathophysiological progression of septic cardiomyopathy and vascular paralysis, as shown in Fig. 8. As mentioned previously, lncRNAs and circRNAs regulate miRNAs by sponging or decoying miRNAs in sophisticated ceRNA networks. MiRNA activation or inhibition leads to the degradation or renaturation of target mRNAs, thereby modulating the transcriptional and translational modification of downstream genes. Based on existing knowledge, lncRNAs *MALAT1, GAS5, ZFAS1, and XIST* and the circRNAs *TLK1 and ANKRD36,* are related to the pathogenesis of septic cardiomyopathy by regulating inflammation, oxidative response, endothelial dysfunction, macrophage polarization, apoptosis and pyroptosis, along with metabolic energy impairment and ERS. Moreover, the lncRNAs *LUADT1, HULC, and circRNA-0044073* are involved in septic vascular paralysis by modulating endothelial cell apoptosis and the inflammatory cytokine cascade.

It is worth noting that *MALAT1, GAS5* and NF-κB are highly expressed and *XIAP* is expressed at low levels both *in vivo* and *in vitro* in SCVDs, which may be the key molecules for sepsis-induced cardiovascular toxicity. Among them, *miR-146*, *miR-150*, *miR-150-5p*, and *miR-26a* are common vector sponges between *MALAT1* and NF-κB, and negatively regulate cardiotoxicity [36, 38, 57, 58]. Meanwhile, *miR‑26a*, *miR-214*, and *miR‑449* are common targets of *GAS5* and negatively regulate the expression of *HMGB1/NF-κB* to relieve cardiotoxicity [27, 28, 29]. In addition, *MALAT1* mediates *miR-23a/MCEMP1*, *miR-26a-5p/RCAN2*, and *hsa-miR-346/SMAD3* to aggravate LPS-induced inflammation and apoptosis [37, 55, 56], while *CYTOR/miR-24*, *KCNQ1OT1/miR-192-5p*, and *SNHG1/miR-181a-5p* jointly target *XIAP* to promote proliferation and inhibit apoptosis [25, 33, 45].

Understanding the ceRNA networks contributes to further understanding of the molecular mechanisms of SCVDs and is expected to seek breakthroughs in the ncRNA-dependent treatment of septic CVDs. Based on the mechanisms discovered, it may be possible to focus on positively or negatively regulating key lncRNAs or circRNAs to prevent the pathological progression of SCVDs at the translational and posttranscriptional levels. Simultaneously, it might also be a good idea to restore or block certain miRNA functions to modulate target mRNAs for subsequent biological effects. On the other hand, previous studies partially explained the molecular mechanisms underlying the biological effects of SCVDs, suggesting that we can prevent cardiovascular dysfunction in sepsis by suppressing inflammation and oxidative stress, restoring endothelial and mitochondrial function, and inhibiting apoptosis and pyroptosis [8, 72, 80, 81, 86, 88].

Despite the great potential of the ceRNA network as a therapeutic target and diagnostic biomarker for SCVDs, there are numerous limitations affecting its clinical applications. In the first place, a great quantity of animal and cell experiments have demonstrated that ceRNA axes play important regulatory roles in SCVD models *in vivo* and *in vitro*, but there is a lack of further confirmation in large-sample clinical trials, especially in multicenter prospective studies. In addition, the clinical characteristics of sepsis are complicated and involve multiple systems and organs. Using only a certain ncRNA for early diagnosis and prognostic monitoring cannot fully reflect the severity and outcome of SCVDs, and a comprehensive evaluation should be combined with disease progression and treatment feedback. Moreover, the ceRNA network is not a simple one-to-one linear axis but a crisscross and interrelated map with multiple targets and pathways. The same lncRNA or circRNA can act on different miRNAs, and different miRNAs can regulate the same target mRNA, thereby mediating different biological effects. Consequently, a satisfactory therapeutic effect cannot be obtained through specific ncRNA-targeted therapy, and multiorgan support and early bundled treatment are needed. Although there are still limitations in the transformation from bench to bedside, it is undeniable that revealing the ceRNA network is beneficial for deciphering the pathogenic mechanism of SCVDs and providing directions for further clinical diagnosis and treatment.

## Abbreviations

AQP1, Aquaporin protein 1; ATP, Adenosine triphosphate; *ATF6*, Activating transcription factor 6; CeRNAs, Competitive endogenous RNAs; CircRNAs, Circular RNAs; CVDs, Cardiovascular diseases; CHF, Chronic heart failure; *CHRF*, Cardiac hypertrophy related factor; *CRNDE*, Colorectal neoplasia differentially expressed; DAMPs, Damage-associated molecular patterns; *DANCR*, Discrimination antagonizing ncRNA; DLR, Dual-luciferase reporter gene assay; ER, Endoplasmic reticulum; GSH/GSSG, Glutathione reduction/oxidation; HCAECs, Human primary coronary artery endothelial cells; HMECs, Human microvascular endothelial cells; *HULC*, Highly upregulated in liver cancer; HUVAMCs, Human vascular smooth muscle cells; HUVECs, Human umbilical vein endothelial cells; *IRE1α*, inositol-requiring enzyme 1α; LncRNAs, Long noncoding RNAs; LPS, Lipopolysaccharide; *LUCAT1*, Lung cancer-related transcript 1; *LUADT1*, Lung adenocarcinoma transcript 1; *MALAT1*, Metastasis-associated lung adenocarcinoma transcript 1; MAMs, Mitochondria-associated membranes; MDA, Malondialdehyde; mRNA, Messenger RNA; MMP, Mitochondrial membrane potential; miRNAs, microRNAs; *MIAT*, Myocardial infarction associated transcript; MRE, miRNA response element; NcRNAs, Non-coding RNAs; *NEAT1*, Nuclear paraspeckle assembly transcript 1; ox-LDL, Oxidized low-density lipoprotein; PA, Palmitic acid; PAMPs, Pathogen-associated molecular patterns; PERK, Pancreatic endoplasmic reticulum kinase; PRRs, Pattern recognition receptors; *PVT1*, Plasmacytoma variant translocation 1; RIP, RNA-binding protein immunoprecipitation; *RMRP*, RNA component of mitochondrial RNA processing endoribonuclease; RNA-FISH, RNA fluorescent in situ hybridization; ROS, Reactive oxygen species; RT, Ricin toxin; SCVDs, Sepsis-related cardiovascular diseases; SFA, Saturated fatty acid; *SNHG1*, Small nucleolar RNA host gene 1; *SNHG16*, Small nucleolar RNA host gene 16; SOD, Superoxide dismutase; siRNAs, Small interfering RNAs; SIRS, Systemic inflammatory response syndrome; *TTN-AS1*, TTN antisense RNA 1; *TXNIP*, Thioredoxin-interacting protein; *XIST*, X-inactive specific transcript; *ZFAS1*, Zinc finger antisense 1.
